# ﻿A new species of *Cinnamomum* (Lauraceae) from southwestern China

**DOI:** 10.3897/phytokeys.202.76344

**Published:** 2022-07-21

**Authors:** Zhi Yang, Chaoyi Deng, Lulu Wang, Qiming Ban, Yong Yang

**Affiliations:** 1 College of Biology and the Environment, Nanjing Forestry University, 159 Longpan Rd., Nanjing 210037, China Nanjing Forestry University Nanjing China; 2 Karst Region Development Institute of Southwestern Guizhou, 14 Ruijinbei Rd., Xingyi City 562400, China Karst Region Development Institute of Southwestern Guizhou Xinyi China; 3 Forestry Bureau of Wangmo County 552300, China Forestry Bureau of Wangmo County Wangmo County China

**Keywords:** Guizhou, morphology, sect. *Camphora*, taxonomy

## Abstract

Field investigations in Guizhou, China, in 2020 resulted in the discovery of an unknown species of Lauraceae. Morphological studies revealed that it is a new species of CinnamomumSchaeff.sect.Camphora Meisn., based on the large terminal buds, and alternate leaves with pinnate veins. It is distinguished from other species of sect. Camphora by the rather large perulate terminal buds with numerous bracts, larger flowers, oblong-elliptic linear tepals twice as long as the stamens, and the deep cup-shaped fruiting cupule. It is here described and illustrated as a new species, *Cinnamomumguizhouense* C.Y.Deng, Zhi Yang et Y.Yang. A key to distinguish it from related species in the same area is provided. In addition, we list the new species as Critically Endangered (CR), and suggest to conduct *ex situ* conservation, collect seeds and plant the species in botanic gardens.

## ﻿Introduction

*Cinnamomum* Schaeff. (Schaeffer 1760: 74; Lauraceae) is known for the spice, cinnamon, derived from the bark of *Cinnamomumaromaticum* Nees (Nees von Esenbeck 1831: 74), which is well known worldwide (Rohwer 1993). For a long time, the genus was circumscribed to contain and ca. 350 species and to have a pan-Pacific distribution (Rohwer 1993; Lorea-Hernández 1996; van der Werff 2001). Recent phylogenetic and taxonomic studies have transferred the Neotropical species of *Cinnamomum* to other genera so that *Cinnamomum* is now treated as being restricted to Tropical/Subtropical Asia and Oceania (Huang et al. 2016; Rohde et al. 2017; Zeng et al. 2021). Two sections have been recognized in *Cinnamomum*, sect. Camphora Meisn. (Meissner 1864: 24) and sect. Cinnamomum. Sect. Camphora differs from sect. Cinnamomum in having perulate terminal buds (vs. naked terminal buds), pinnately veined alternate leaves (vs. tripliveined opposite/subopposite leaves), and domatia usually present in the axils of the lateral leaf veins (vs. lacking domatia in the axils of the lateral leaf veins) (van der Werff 2001; Huang et al. 2016). Sect. Camphora is restricted to the northern hemisphere while sect. Cinnamomum is distributed from eastern Asia to Oceania (Soh 2011). Interestingly, Trofimov and Rohwer (2020) suggest that Cinnamomum is polyphyletic with sect. Camphora being sister to *Sassafras* J.Presl (Presl 1825: 230) and sect. Cinnamomum being sister to *Kuloa* Trofimov & Rohwer (2020: 527). Until there is a modern taxonomic treatment, we accept species of *Cinnamomum* as belonging to a single genus.

Recent botanical investigations to Wangmo County of Guizhou Province, one of the most botanical diverse regions of China, resulted in the discovery of a *Cinnamomum* species with unusual morphology, which led us to conclude that it is a new species for science. We conducted field investigations, estimated the population size and extent/area of occupancy, and collected several flowers and fruits of the plant. Specifically, we carried out morphological studies to answer the following questions: 1) what are the morphological affinities of the new species; and 2) what is the conservation status of the new species?

## ﻿Material and methods

Field investigations were conducted and observations were made during February of 2020 and February of 2022. Specimens were collected, and flowers were fixed in FAA. Fresh and pickled flowers were dissected, observed and measured under a light microscope (GP-M101). Photographs were taken using a Nikon D7100 and a Sony A7M3 camera. We assessed the conservation status of the species by observing the population size and estimating the extent of occurrence (EOO) and the area of occupancy (AOO) and applying IUCN red list categories and criteria and guidelines (IUCN 2012, 2022). Line drawings were prepared manually with a pen and black ink. Line drawings and figures were edited, arranged, and merged using Adobe Photoshop CS2 ver. 9.0 and Adobe Illustrator. The distribution map was generated with ArcGis ver. 10.0.

## ﻿Results

### ﻿Taxonomy

#### ﻿ Cinnamomum guizhouense

Taxon classificationPlantaeLauralesLauraceae

C.Y.Deng, Zhi Yang & Y.Yang sp. nov.

D1E2DAE3-0D1D-5EF6-B864-76C14D598483

urn:lsid:ipni.org:names:77302159-1

Figs 1 , 2

##### Type.

China. Guizhou, Wangmo Co., Jiaona Tw., Babu Village, Liji Sect., 25°21'8"N, 106°17'44"E, elev. 1081 m, 20 Feb 2021, *C.Y. Deng & Q.M. Ban 2021001* (holotype: NF; isotypes: NF, NAS, XIN).

##### Diagnosis.

*Cinnamomumguizhouense* is close to *C.foveolatum* (Merr.) H.W.Li et J.Li (Li et al. 2008: 170) in having leaves that lack domatia in the axils of the lateral leaf veins and in the long fruiting cupule, but differs from the latter by the longer leaves (12–21 cm vs. 9–15 cm in *C.foveolatum*), longer petioles 2–4 cm long (vs. 1–1.3 cm in *C.foveolatum*), longer tepals (3–4 mm long vs. 1.7–2 mm long in *C.foveolatum*), and shorter campanulate or cup-shaped cupules ca. 15 mm long (vs. cupules up to 20 mm long in *C.foveolatum*).

##### Description.

**Figure 1.**
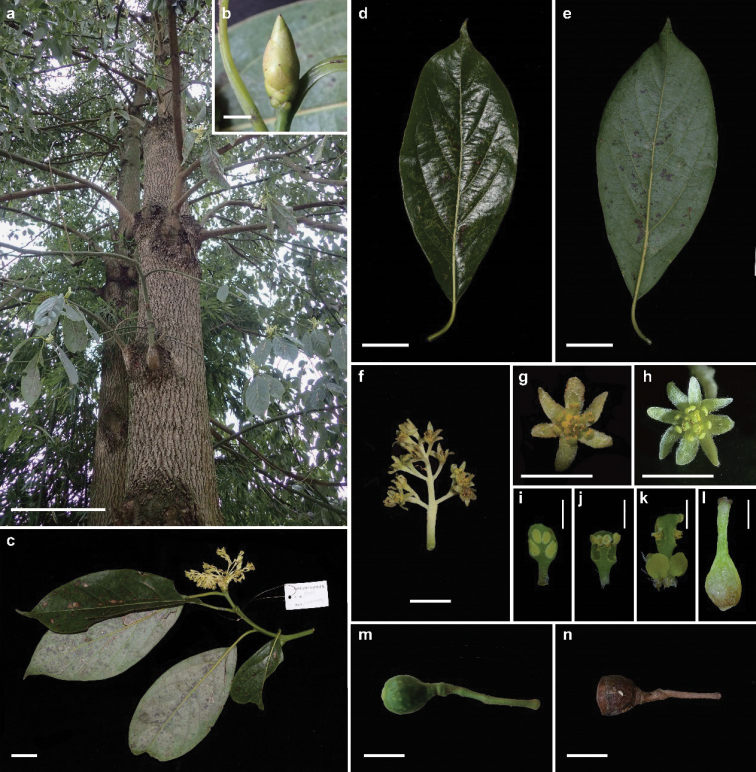
Morphology of *Cinnamomumguizhouense* C.Y. Deng, Zhi Yang et Y. Yang, sp. nov. **a** trunk **b** bud **c** flower branch **d** leaf adaxial surface **e** leaf abaxial surface **f** inflorescence **g** trimerous flower **h** tetramerous flower **i** stamen of first whorl **j** stamen of second whorl **k** stamen of third whorl with two glands at base **l** pistil **m** a young fruit with pedicel and cupule enclosing inner fruit **n** mature infructescence with deep, cup-shaped cupule. Bars: 30 cm (**a**); 1 cm (**b, f–h, m, n**); 3 cm (**c-e**); 1 mm (**i–l**).

**Figure 2.**
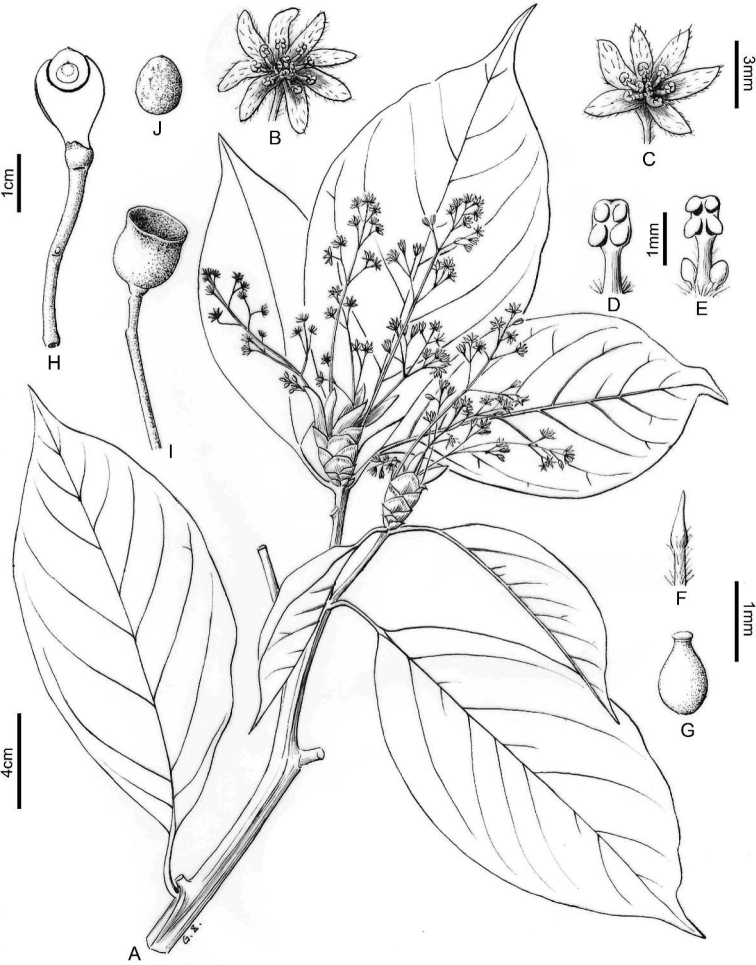
Illustration of morphological characters of *Cinnamomumguizhouense* C.Y. Deng, Zhi Yang & Y. Yang, sp. nov. **A** leafy flowering branch with alternate, elliptic and obovate leaves, large terminal buds and inflorescences **B, C** variation in floral merosity **B** tetramerous flower **C** trimerous flower **D** stamen of first and second whorl **E** stamen of third whorl **F** staminode of fourth whorl **G** pistil **H** longitudinal section of young fruit with pedicel and cupule partially enclosing inner fruit **I** mature infructescence with peduncle and pedicel and deep cup-shaped cupule **J** subglobose seed.

Trees, evergreen, 11–13 m tall, D.B.H. ca. 36 cm (Fig. 1a); crown columnar to pyramid shaped, ca. 3 m in diam. Trunk straight; bark gray, longitudinally fissured. Twigs angular, glabrous, with multiple circular scale scars at base of twigs. Terminal buds prominent, to 2.5 cm long, 1.5 cm in diam. (Fig. 1b); bracts many (to 15), obovate to oblanceolate, apex obtuse, acute to cuspidate, abaxially pubescent, adaxially glabrous, margin ciliate. Leaves alternate; petioles glabrous, channeled abaxial, 2–4 cm long (Figs 1c–e, 2a); blade coriaceous, elliptic to obovate-elliptic, 6–9×12–21 cm, base acute to cuneate, more or less decurrent, apex acuminate, pinnately veined, secondary veins 5–7 pairs, midrib and secondary veins immersed or slightly raised on adaxial surface, elevated on abaxial surface; adaxially green, abaxially glabrous and glaucous. Panicles terminal (Figs 1c, 1f, 2a), 7–10 cm long, peduncles, pedicels and tepals pubescent, lateral flowers of ultimate cymes opposite, pedicels 4–6 mm long. Flowers bisexual, receptacle prominent, obconic, ca. 1 mm long, 1 mm in diam.; perianth in two whorls, tepals 6–8, subequal, oblong-elliptic to linear, 3–4 mm long, 1 mm wide, both surfaces pubescent, margin ciliate (Figs 1g, 1h); stamens in three whorls, 3 or 4 in each whorl, 1.5–2 mm long, filaments subequalling anthers, glabrous, each stamen of third whorl with two globose glands at base; anthers yellow, 4–locular, those of outer two whorls introrse, those of third whorl extrorse (Figs 1i–1k, 2d–2e); staminodes of fourth whorl sagittate, filaments pubescent (Fig. 2f); pistil glabrous, ovary ovoid to ellipsoid, 1.2 mm long, ca. 0.8 mm in diam., style glabrous, ca. 1 mm long, stigma peltate when fresh, inconspicuous when dry (Figs 1l, 2g). Infructescences 6–15 cm long. Young fruit together with cupules obovoid-ellipsoid,15–20 mm long, 13–17 mm in diam., 3/4 or more of fruit enclosed in cupule (Fig. 1m). Fruit black when mature, cupulate, cupules campanulate to cup-shaped, ca. 1.5 cm long, 1.5 cm in diam. (Fig. 1n, 2i); pedicels thickened, 4–6 mm in diam.; seeds ellipsoid to subglobose (Fig. 2J), ca. 1 cm long, 8 mm in diam., yellowish-brown, longitudinally ridged. Flowering February; fruiting September and October.

##### Etymology.

The species is named after the province, Guizhou, where it occurs.

##### Distribution.

**Figure 3.**
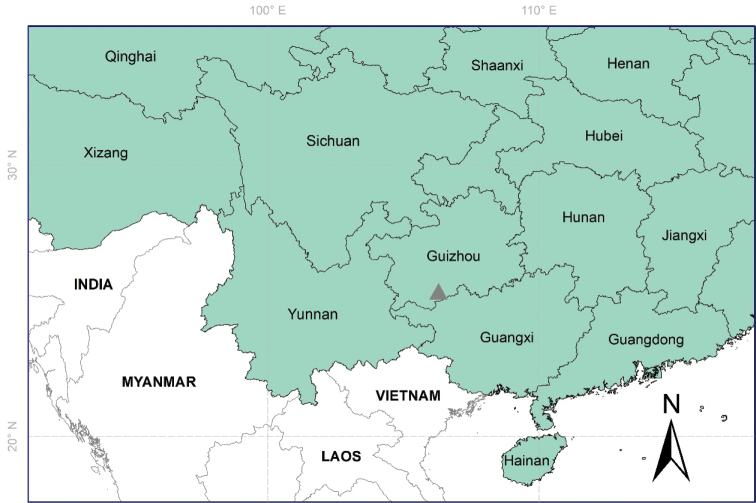
Map showing distribution of *Cinnamomumguizhouense* C.Y. Deng, Zhi Yang et Y. Yang, sp. nov. (gray triangle).

*Cinnamomumguizhouense* is known only from Wangmo Xian, Guizhou Province, southwestern China (Fig. 3).

##### Ecology and habitat.

The new species lives in bamboo (*Phyllostachys* sp.) colonies in acidic soil with mean annual temperature 13–15 °C, annual precipitation 1000–1200 mm. It is heliophilous and lives on western slopes. The species lives near a village road with human disturbance, and the disturbance will not stop unless a new nature reserve is established to conserve the species. The species has an extremely small population, and only two individuals were found in the region. The living individuals occupy an area (EOO, ≈ AOO) ca. 100 m^2^.

##### Conservation.

On four instances of field investigations in the area, we found only two mature plants living within ca. 100 m^2^. We thus designate the species to be Critically Endangered (CR B1+2ab(iii); C1+2a(i); D) according to the IUCN red list categories and criteria (IUCN 2012, 2022), but we acknowledge that further assessments are necessary as new populations are found.

##### Vernacular name.

The local people refer to *C.guizhouense* as Da Mu Jiang Zi (big *Litsea*), which is not in accordance with its formal taxonomic position. Here we name it Guizhou Cinnamon.

##### Additional specimens examined.

China. Guizhou, Wangmo Xian, Jiaona Tw., Babu Village, Liji Section, 25°21'8"N, 106°17'44"E, elev. 1081 m, 20 Feb 2021, *C.Y. Deng & Q.M. Ban 2021002* (NF, XIN), *2021003* (NF, XIN); 21 Oct 2020, *C.Y. Deng et al. 2020102111* (XIN); 20 Oct 2020, *C.Y. Deng & J.B. Xu 2020102802* (XIN); 10 Jun 2021, *C.Y. Deng & Q.M. Ban 2021061001 & 2021061002* (XIN).

## ﻿Discussion

Recent phylogenetic studies have suggested that *Cinnamomum* comprises two groups that largely, but not strictly, correspond to two sections of the genus (Huang et al. 2016; Liu et al. 2021; Zeng et al. 2021). Huang et al. (2016) reported that *C.saxatile* H.W.Li (Li 1975: 44) and *C.longipetiolatum* H.W.Li (Li 1975: 47) and an unidentified species C. sp. C684 actually belong to sect. Cinnamomum and not to sect. Camphora as traditionally circumscribed. *Cinnamomumguizhouense* belongs to sect. Camphora according to our plastome phylogeny (unpubl. data). This finding was corroborated by the large perulate buds and pinnately veined, alternate leaves of *C.guizhouense*.

*Cinnamomumguizhouense* is characterized by the large perulate terminal buds, flowers with tepals twice as long as the stamens, and deep fruiting cupules. This unique combination of morphological characteristics distinguishes *C.guizhouense* from all other species of sect. Camphora (Li et al. 1982, 2008). We made a morphological comparison of *C.guizhouense* with other species of sect. Camphora in Guizhou (Table 1). *Cinnamomumguizhouense* is similar to *C.foveolatum* in the deep fruiting cupules, but differs from the latter in the longer leaves 12–21 cm (vs. leaves 9–15 cm long in *C.foveolatum*), oblong-elliptic to linear tepals 3–4 mm long (vs. ovate to broadly ovate tepals 1.7–2 mm long in *C.foveolatum*), longer stamens 1.5–2 mm long (vs. stamens 1.2–1.4 mm long in *C.foveolatum*) and shorter fruiting cupules ca. 15 mm (vs. fruiting cupules ca. 20 mm in *C.foveolatum*). *Cinnamomumguizhouense* resembles *C.saxatile* in the tepals 3–4 mm long (Li et al. 1982, 2008), but differs in having shorter stamens 1.5–2 mm (vs. tepals subequalling stamens, ca. 4 mm in *C.saxatile*). *Cinnamomumsaxatile* was ascribed to sect. Camphora because of its alternate, pinnately veined leaves (Li et al. 1982, 2008), but has been demonstrated to belong to sect. Cinnamomum according to the leaf anatomy and phylogenetic evidence (Huang et al. 2016; Zeng et al. 2021). *Cinnamomumguizhouense* was close to *C.camphora* (L.) J.Presl (Presl 1825: 47) and *C.bodinieri* H.Lév. (Léveillé 1912: 369) in sect. Camphora in our plastome phylogeny (unpubl. data), but differed from *C.camphora* and *C.bodinieri* in the absence of domatia in the axils of the leaves, much longer tepals (3–4 mm vs. 1.5–2 mm in *C.camphora* and *C.bodinieri*), and the deep, cup-shaped fruiting cupules (vs. flat cupules in *C.camphora* and *C.bodinieri*). A key to these closely related species is provided. Moreover, we found that floral merosity of our new species is variable. Variation of floral merosity, including tetramerous flowers, has also been recorded in *Beilschmiediaappendiculata* (C.K.Allen) S.K.Lee & Y.T.Wei (Li et al. 1979: 65) and *Syndiclis* spp (Zeng et al. 2017, 2021), and also in *Caryodaphnopsis* sp. (pers. observ.). The variable merosity of flowers in the family may have been caused by change of selection pressure. Further studies are necessary to figure out what kind of selection pressure works on the change of floral merosity. Our finding not only increases species diversity of *Cinnamomum* in Guizhou and China but also expands our knowledge of the morphological diversity of *Cinnamomum*.

**Table 1.** Morphological comparison of species of Cinnamomumsect.Camphora from Guizhou, China.

Species	Leaf size (cm)	Lateral veins	Petiole length (cm)	Inflorescence pubescence	Tepal shape	Tepal length (mm)	Stamen length (mm)	Cupule length (mm)
***C.bodinieri***	8−17 × 3−10	4−6	2−3	glabrous	ovate	1.2	1 or a little longer	/
***C.camphora***	6−12 × 2.5−5.5	1−5(−7)	2−3	glabrous or gray- to yellow-brown puberulent	elliptic	2	2	5
***C.foveolatum***	9−15 × 3−5.5	6−8	1−1.3	sparsely villous	outer ones ovate, inner ones broadly ovate	outer ones ca. 2 × 1.1, inner ones ca. 1.7 × 1.2	1.2−1.4	20
***C.glanduliferum***	6−15 × 4−6.5	4 or 5	1.5−3.5	glabrous	broadly ovate	2 × 1.7	1.4−1.6	10
***C.guizhouense***	**12−21 × 6−9**	**5−7**	**2−4**	**pubescent**	oblong-elliptic to linear	**3−4**	**1.5−2**	**15**
***C.micranthum***	7.5−10 × 4−6	4 or 5	2−3	subglabrous or slightly puberulent	narrowly ovate	1.3	1	9
***C.migao***	4.5−16 × 2.5−7	4 or 5	1.3−3	pubescent	/	/	/	12
***C.parthenoxylon***	6−12 × 3−6	4 or 5	1.5−3	glabrous	narrowly elliptic	2 × 1.2	1.5−1.7	10 mm or less
***C.rufotomentosum***	15−16.5 × 4−5	4−6	2−2.9	reddish brown tomentose	/	/	/	/
***C.saxatile***	5−13 × 2−5	5−7	0.5−1.5	brownish puberulent	ovate	3	4−4.5	shallow

*Morphological data was extracted from *Flora of China* (Li et al. 2008).

We conducted field investigations on *C.guizhouense* on four occasions and found only two individuals in the area, suggesting that the species has an extremely small population. In addition, the new species lives near a village with human disturbance, the living habitat of *C.guizhouense* has not been improved and deterioration continues. We thus suggest to plan an *ex situ* conservation strategy for the new species, collect seeds and plant the species in botanic gardens.

### ﻿Key to similar species in the same region

**Table:** 

1a	Tepals of flowers short, 1−2 mm long	**2**
1b	Tepals of flowers 3−4 mm long	**4**
2a	Fruiting cupule up to 20 mm long	***C.foveolatum***
2b	Fruiting cupule ca. 5 mm long	**3**
3a	Leaves 8−17 cm long; tepals ovate, 1.2 mm long; stamens 1 mm long	***C.bodinieri***
3b	Leaves 6−12 cm long; tepals elliptic, 2 mm long; stamens 2 mm long	***C.camphora***
4a	Buds 2−5 mm long; domatia present in the axils of lateral leaf veins; panicles 3−6 cm long; stamens 4−4.5 mm long; fruiting cupule shallow, 5−6.5 mm in diam	***C.saxatile***
4b	Buds to 25 mm long; domatia absent in the axils of lateral leaf veins; panicles 7−10 cm long; stamens 1.5−2 mm long; fruiting cupule deep to 15 mm in diam	***C.guizhouense***

## Supplementary Material

XML Treatment for Cinnamomum guizhouense
